# The Establishment and Dynamic Properties of a New 4D Hyperchaotic System with Its Application and Statistical Tests in Gray Images

**DOI:** 10.3390/e22030310

**Published:** 2020-03-10

**Authors:** Lina Ding, Qun Ding

**Affiliations:** 1 Electronic Engineering College, Heilongjiang University, Harbin 150080, China; 1141738@s.hlju.edu.cn; 2 Electrical Engineering College, Suihua University, Suihua 152061, China

**Keywords:** hyperchaotic system, image encryption, entropy test, statistical tests

## Abstract

In this paper, a new 4D hyperchaotic system is generated. The dynamic properties of attractor phase space, local stability, poincare section, periodic attractor, quasi-periodic attractor, chaotic attractor, bifurcation diagram, and Lyapunov index are analyzed. The hyperchaotic system is normalized and binary serialized, and the binary hyperchaotic stream generated by the system is statistically tested and entropy analyzed. Finally, the hyperchaotic binary stream is applied to the gray image encryption. The histogram, correlation coefficient, entropy test, and security analysis show that the hyperchaotic system has good random characteristics and can be applied to the gray image encryption.

## 1. Introduction

Since Lorenz [1] discovered the first three-dimensional chaos model, chaos theory has grown with the development of computer science. Chaos is an unpredictable and random motion in deterministic dynamical systems due to its sensitivity to initial values. The certainty of a dynamic system is a concept defined in mathematics, which means that the state of the system at any time can be determined by the initial state of the system. Although the motion state of the deterministic dynamic system at any time can be calculated according to the initial state and motion law, the measurement of the initial state and data cannot be completely accurate. Even a slight difference will lead to a very large error in the predicted results, to an unpredictable degree. In recent years, as chaotic systems have many advantages in encryption, such as ergodicity, unpredictability, pseudo-randomicity, and high sensitivity to parameters and initial values [2], image encryption based on chaos has become a research hotspot. Aside from image encryption based on chaotic systems, there are many representative methods such as: based on one-time keys, bit-level permutation, DNA rule, matrix, and semi-tensor product theory [3,4].

At present, research on 1D chaos, such as Logistic mapping [5,6,7]; 2D chaos, such as Henon mapping [8,9,10]; and 3D chaos, such as Rossler chaotic attractor [11,12,13], Chua [14,15,16], and Chen [17,18,19], have been very extensive and mature. With the development of chaos theory, many people began to study high-dimensional chaotic attractors, such as 4D chaotic attractor subsystems [20,21,22,23], 5D chaotic attractor subsystems [24,25,26,27], and 6D chaotic attractor subsystems [28]. In recent years, fractional-order chaotic systems [29,30,31], hidden attractors [32,33,34], and chaotic systems with co-existing attractors [35,36] have also been extensively studied. In ordinary three-dimensional chaotic attractors, linear or nonlinear state feedback controllers can generate different kinds of four-dimensional chaotic systems. The 4D hyperchaotic system has better computational complexity and two or more positive Lyapunov exponents [37,38].

Recently, many scholars have generated many new chaotic systems on the basis of studying the Lorenz chaotic system, which are collectively referred to as Lorenz type hyperchaotic systems [39,40]. These new systems are applied to many aspects, such as chaotic synchronization [41], image encryption [8,12,13,19], stream cryptography [42,43], and so on. 

The chaos-based image encryption systems are usually applied to generate chaotic stream ciphers for exchanging the positions or values of the pixels in the original images. A 2D chaotic Arnold cat map was used to generate a 3D cat map, which then was used in image encryption [44]. The results show that the scheme is fast and safe. The authors of [45] applied Henon mapping to the image encryption scheme, and proved that the encryption method could resist selective plaintext attack, etc. The authors of [46] proposed an image encryption scheme based on Logistic mapping, and the authors of [47] proposed an image encryption scheme based on the 3D chaotic system. The above image encryption methods using chaotic systems are based on low-dimensional chaotic systems with at most one positive Lyapunov exponent, which have many advantages, such as simple format, few control parameters, and ease of implementation. However, low-dimensional chaotic systems are vulnerable to attack. If low-dimensional chaotic systems are changed into high-dimensional chaotic systems, the encryption will be more effective. Lyapunov exponent (LE) is an effective method to measure chaotic systems. If a chaotic system has two or more positive LEs, it can be called a hyperchaotic system, which usually has a larger key space and much higher security in encryption schemes [37,38]. As the chaotic systems with four dimensions or more have two or more Lyapunov exponents and better dynamic characteristics, the application to image encryption will have better practical effects [37,38]. The authors of [48] presented a novel approach that uses a hyperchaotic system, Pixel-level, and DNA-level diffusion. The authors of [49] proposed a new image encryption method based on matrix semi-tensor product theory and hyperchaotic Lorenz. The research above shows that the application of hyperchaotic system encryption has become an important trend.

The main contributions of this paper are shown as follows: (1) A new 4D hyperchaotic system is generated, and the dynamic properties of the attractor such as phase space, local stability, poincare section, periodic attractor, quasi-periodic attractor, chaotic attractor, bifurcation diagram, and Lyapunov index are analyzed; (2) Then the new hyperchaotic system is normalized and binary serialized, and the binary hyperchaotic stream generated by the system is statistically tested and entropy analyzed; (3) The hyperchaotic binary stream is applied to the gray image encryption; (4) The histogram, correlation coefficient, entropy test, and security analysis show that the hyperchaotic system has good random characteristics and can be applied to the gray image encryption.

The main advantages of this paper are shown as follows: (1) A new 4D hyperchaotic system based on Lorenz is proposed and analyzed; (2) The hyperchaotic system with two positive LEs is much more random, which is then used to generate sequences for the encryption operations; (3) The new hyperchaotic system in this paper is obtained by adding a new variable, w, and a feedback controller, $-dx^{3}$, to the classical Lorenz chaotic attractor system. In this way, an equilibrium point curve exists in the system which is a new phenomenon in the system. 

In this paper, a new 4D hyperchaotic system is proposed by studying Lorenz-type hyperchaotic system, and the corresponding dynamic properties, such as Lyapunov exponent, phase space diagram, poincare section diagram, and local stability are studied. The method of normalization and binarization is applied to the encryption of gray image. Finally, the entropy test and security analysis of image encryption are carried out. 

The rest of this paper is organized as follows: Section 2 introduces a new 4D hyperchaotic system based on Lorenz system with two positive LEs. In Section 3, analyses of the dynamic properties are done, such as judgment of local stability, Poincare section diagram, periodic attractor, etc. In Section 4, normalization and quantization are done. Furthermore, NIST tests, permutation entropy, and approximate entropy are completed to test the time series of the hyperchaotic system. In Section 5, the hyperchaotic system is used in image encryption. Then, the encryption effect and security are tested by correlation coefficient analysis, information entropy, differential attack, etc. Finally, the paper is summarized in Section 6.

## 2. A New Hyperchaotic System 

In 1963, a representative Lorenz equation in chaotic attractors was proposed [1]. The differential expression of this equation is shown as follows Equation (1):

$$\dot{x}=\sigma (y-x),$$

(1) $$\dot{y}=\alpha x-y-xz,$$

$$\dot{z}=xy-\beta z.$$

**Figure 1:**
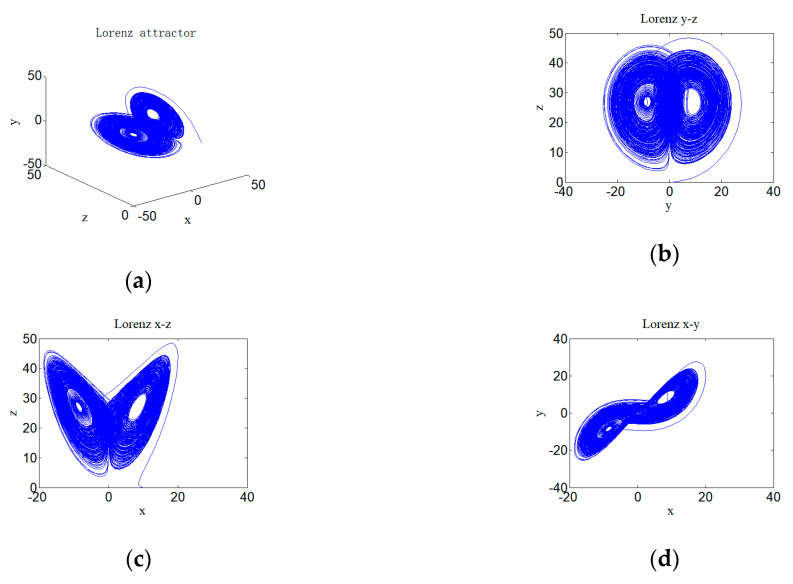
Chaotic Lorenz attractor and its phase diagrams with σ=10, α=28, and β=8/3: (**a**) Lorenz attractor; (**b**) Lorenz attractor on y–z plane; (**c**) Lorenz attractor on x–z plane; and (**d**) Lorenz attractor on x–y plane.

The equation set is a third-order system of ordinary differential equations, and each variable in the equations does not obviously contain time t, so the equation set is called an autonomous system. Its parameters, σ, α, and β, are all constants greater than zero. When the parameters of this equation are taken as σ=10, α=28, and β=8/3, the system presents chaotic attractor state, namely the classical Lorenz attractor, and its phase space is shown in Figure 1. The numeric computation method used to compute the chaotic system or hyperchaotic system is the 4th order Runge–Kutta method.

The new hyperchaotic system in this paper is obtained by adding a new variable, w, and a feedback controller, $-dx^{3}$, to the classical Lorenz chaotic attractor system. The new hyperchaotic system is expressed as follows in Equation (2):

$$\dot{x}=a(y-x),$$

$$\dot{y}=bx-y-xz+w,$$

(2) $$\dot{z}=x^{2}-cz,$$

$$\dot{w}=w-dx^{3}.$$

**Figure 2:**
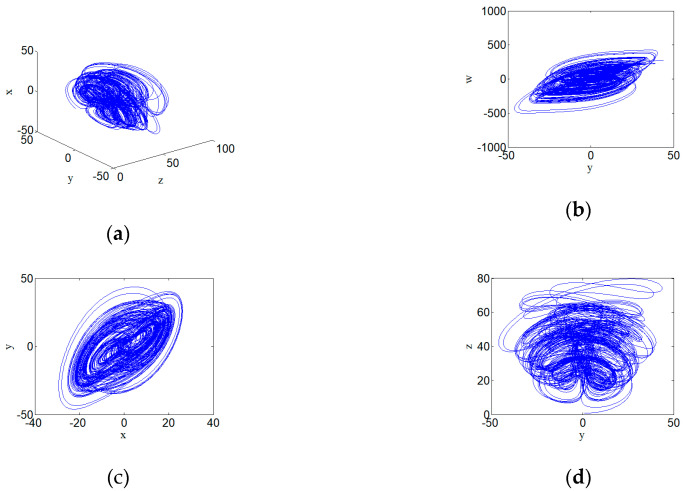
Hyperchaotic attractors and the phase diagrams with parameters a=24, b=25, c=3, and d=0.5: (**a**) hyperchaotic attractor; (**b**) hyperchaotic attractor on y–w plane; (**c**) hyperchaotic attractor on x–y plane; and (**d**) hyperchaotic attractor on y–z plane.

Where a, b, c, and d are all constants greater than zero. Let a∈[20,30]. There are two positive LEs over a wide range of parameters, which implies that the system here is hyperchaotic, as shown in Figure 9b. Fix the parameter a=24, then set the parameters of the system with a=24, b=25, c=3, d=0.5, and initial condition (1,1,1,1). The system can present the state of a hyperchaotic system, as shown in Figure 2. The Lyapunov exponent corresponding to the hyperchaotic system is shown as follows:

$$\lambda_{1}=0,\lambda_{2}=2.5274,\lambda_{3}=2.1036,\lambda_{4}=-16.3014.$$

The divergence of the hyperchaotic system can be expressed as Equation (3):

(3) $$\nabla V=\frac{\partial \dot{x}}{x}+\frac{\partial \dot{y}}{y}+\frac{\partial \dot{z}}{z}+\frac{\partial \dot{w}}{w}=-(a+c)<0,$$

According to Equation (3), when a+c>0, the hyperchaotic system is a dissipative system. 

**Figure 3:**
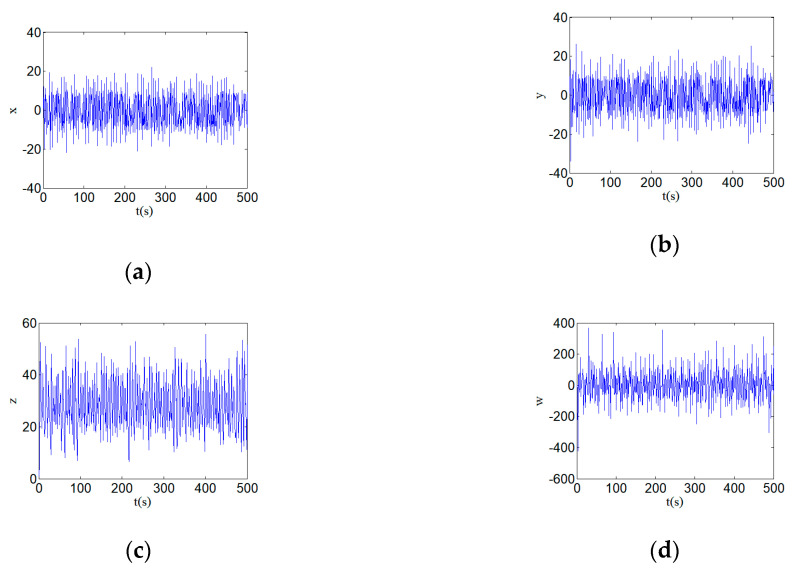
Time series diagrams of a hyperchaotic system with parameters a=24, b=25, c=3, and d=0.5: (**a**) time series x; (**b**) time series y; (**c**) time series z; and (**d**) time series w.

Figure 2 shows the new hyperchaotic attractors and the phase diagrams with parameters a=24, b=25, c=3, and d=0.5. (a) shows the hyperchaotic attractor, (b) shows the hyperchaotic attractor on y–w plane, (c) shows the hyperchaotic attractor on x–y plane, (c) shows the hyperchaotic attractor on y–z plane. The time series diagrams of the phases x, y, z, and w of the hyperchaotic system is shown in Figure 3. Figure 3 shows the time series diagrams of the hyperchaotic system, and it can be seen that the sequences have good randomness.

## 3. Analysis of Dynamic Properties

### 3.1. Subsection Equilibrium Curve

Obviously, according to system (2), an equilibrium point curve exists in the system, and the equation of the equilibrium point curve can be expressed as Equation (4):

(4) $$\left\{(x,y,z,w)\in R^{4}|y=x,z=\frac{1}{c}x^{2},w=dx^{3}\right\},$$

**Figure 4:**
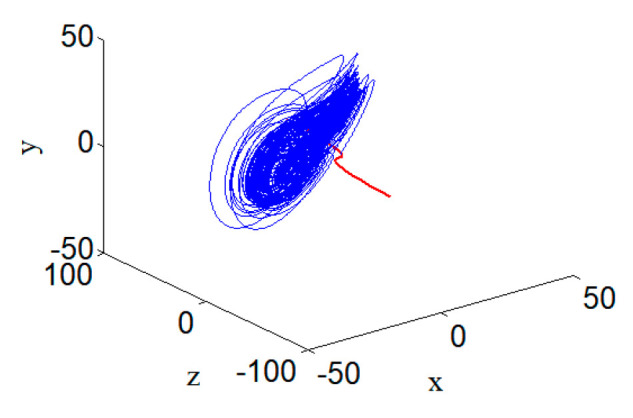
Hyperchaotic attractor and its equilibrium curve with parameters a=24, b=25, c=3, and d=0.5.

The position relationship between this curve and system (1) is shown in Figure 4, which shows the hyperchaotic system attractor and its equilibrium curve in red.

### 3.2. Judgment of Local Stability

It is easy to know from Equation (2) that when $\frac{c(1-b)}{dc-1}<0$ there is only one equilibrium point, O(0,0,0,0), for the system. When $\frac{c(1-b)}{dc-1}\geq 0$, the system has three balance points: O(0,0,0,0) and $O_{1,2}(\pm \frac{c(1-b)}{dc-1},\pm \frac{c(1-b)}{dc-1},c{\left(\frac{1-b}{dc-1}\right)}^{2},\pm dc^{2}{\left(\frac{1-b}{dc-1}\right)}^{3})$.

Let k∈R, according to Equation (2), the point $A=(k,k,k^{2}/3,0.2k^{3})$ is on the equilibrium point curve. Therefore, the Jacobian matrix at the equilibrium point, A, of the hyperchaotic system can be obtained as follows:

(5) $$J=\left(\begin{array}{cccc} -a & a & 0 & 0\\ b-k^{2}/3 & -1 & -k & 1\\ 2k & 0 & -c & 0\\ -3dk^{2} & 0 & 0 & 1 \end{array}\right),$$

Then the characteristic equation can be obtained:

(6) $$\lambda^{4}+g_{1}\lambda^{3}+g_{2}\lambda^{2}+g_{3}\lambda^{1}+g_{4}=0,$$

The coefficients in the equation are:

$$g_{1}=a+c,$$

$$g_{2}=ac-ab+ak^{3}/3-1$$

(7) $$g_{3}=-a-c+ab-abc+\frac{5}{3}ak^{2}+\frac{1}{3}ack^{2}+3adk^{2}$$

$$g_{4}=-ac+abc+3acdk^{2}-\frac{1}{3}ack^{2}-2ak^{2}$$

According to the Routh–Hurwitz criterion, if the coefficients of the first column in the Routh array table are all positive, the system is stable. That is, all the roots of the characteristic equation are located in the left half plane of the root plane and have negative real parts. From the criterion, it can be known that the four coefficients $g_{1}$, $g_{2}$, $g_{3}$, and $g_{4}$ should be positive, and $g_{1}g_{2}-g_{3}>0$ and $g_{1}g_{2}g_{3}-g_{3}{}^{2}-g_{4}g_{1}{}^{2}>0$.

### 3.3. Poincare Section Diagram

**Figure 5:**
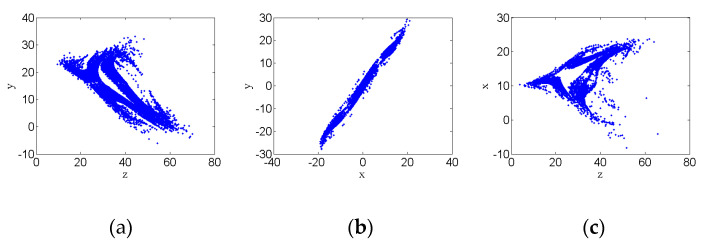
Poincare section of hyperchaotic system attractors with parameters a=24, b=25, c=3, and d=0.5: (**a**) Poincare section on z–y plane; (**b**) Poincare section on x–y plane; and (**c**) Poincare section on z–x plane.

The complex motion of a chaotic system is described by the Poincare section method proposed in the 19th century. It can be known from the section diagram that if only one fixed point or relatively few discrete points are shown on the section, the motion can be judged as periodic motion. When the Poincare section shows a closed curve, the motion can be judged as quasi-periodic motion. When there are dense points in the section, the motion can be judged as complex chaotic motion. For this hyperchaotic system, the Poincare section is also used to observe its motion, which is shown in Figure 5. Figure 5 shows the Poincare section of the hyperchaotic system. It can be seen that the Poincare section presents dense spots in patches, which means the system is chaotic.

### 3.4. Periodic Attractor

**Figure 6:**
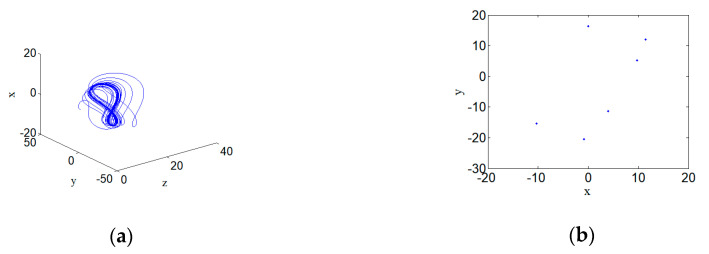
Poincare section of periodic attractor for hyperchaotic systems with a=4, b=5, c=3, and d=0.5: (**a**) Periodic attractor, and (**b**) Poincare section.

When the hyperchaotic system (2) has the parameters a=4, b=5, c=3, and d=0.5, and its initial conditions are defined as (1,1,1,1), the periodic attractor appears in the phase space of the system. Its 3D phase space projection diagram and the corresponding Poincare section diagram are shown in Figure 6. Figure 6 shows the Poincare section of the hyperchaotic system. It can be seen that the Poincare section presents a few discrete points, which means the system is periodic.

### 3.5. Quasi-Periodic Attractors

**Figure 7:**
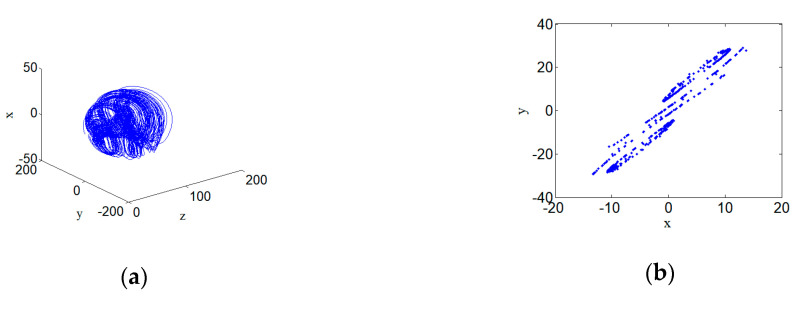
Poincare section of quasi-periodic attractor for hyperchaotic systems with a=7, b=25, c=3, and d=0.5: (**a**) Quasi-periodic attractor, and (**b**) Poincare section.

When the hyperchaotic system (2) selects parameters a=7, b=25, c=3, and d=0.5, and its initial conditions are defined as (1,1,1,1), the quasi-periodic attractor appears in the phase space of the system, and its 3D phase space projection diagram and the corresponding Poincare section diagram are shown in Figure 7. Figure 7 shows the Poincare section of the hyperchaotic system. It can be seen that the Poincare section presents closed circles, which means the system is quasi-periodic.

### 3.6. Chaotic Attractor

**Figure 8:**
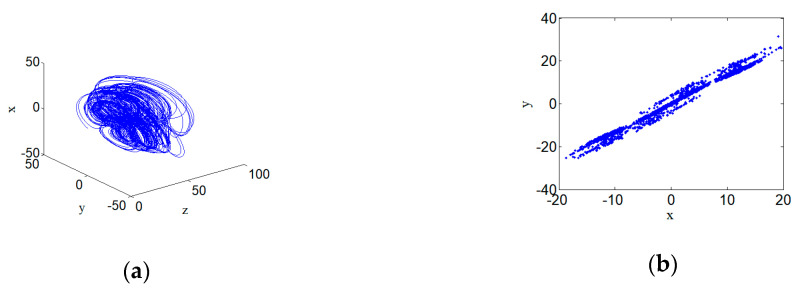
Poincare section of chaotic attractor for hyperchaotic systems with a=14,b=25,c=3 and d=0.5: (**a**) Chaotic attractor, and (**b**) Poincare section.

When system (2) selects parameters a=14, b=25, c=3, d=0.5 and its initial conditions are defined as (1,1,1,1), then the chaotic attractor appears in the phase space of the system. Its 3D phase space projection diagram and the corresponding Poincare section diagram are shown in Figure 8. The Lyapunov exponents corresponding to the chaotic attractor are shown below. Figure 8 shows the Poincare section of the chaotic state of the new hyperchaotic system. 

### 3.7. Bifurcation and Lyapunov Exponent 

**Figure 9:**
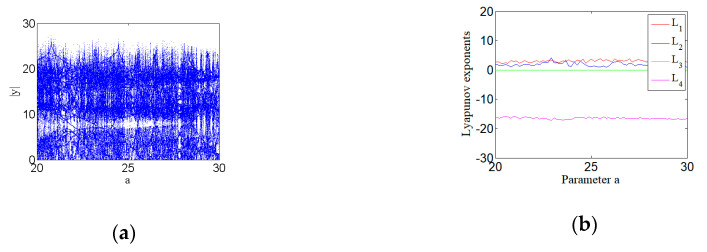
Bifurcation diagram and Lyapunov exponent spectrum of hyperchaotic system with a∈[20,30], b=25, c=3, and d=0.5: (**a**) Bifurcation diagram, and (**b**) Lyapunov exponent spectrum.

When a∈[20,30], the change of the bifurcation diagram and Lyapunov exponent spectrum of the system with changes of parameter a are shown in Figure 9. Figure 9 shows the bifurcation diagram and Lyapunov exponent spectrum of the hyperchaotic system. It can be seen that bifurcation diagram is in a chaotic state in a∈[20,30], and there are two positive LEs, which means that it is a hyperchaotic system.

## 4. Normalization and Quantization

In order to put the hyperchaotic system into use, normalization and quantization are done. The time series after normalization and quantization are tested.

### 4.1. Normalization Treatment

**Figure 10:**
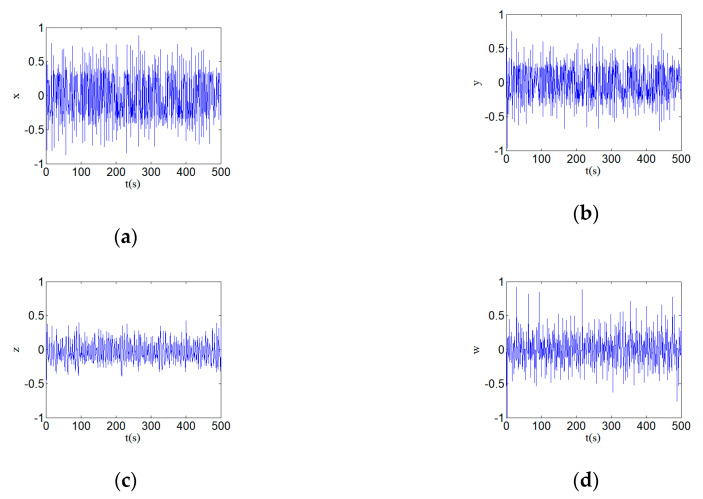
Streams of the normalized hyperchaotic system: (**a**) time series x; (**b**) time series y; (**c**) time series z; and (**d**) time series w.

In order to facilitate the data processing of the hyperchaotic system, the normalization is carried out first. In this paper, the time series data of four output signals, x, y, z, and w, are mapped to the interval [−1,1] and then quantified. The stream of the normalized hyperchaotic system is shown in Figure 10. Figure 10 shows the time series are normalized into the interval [−1,1].

### 4.2. Quantization

For the above hyperchaotic system, it must be converted into binary stream. Here, the quantization function expression is set as Q[x,y,z,w], and the definition is shown as follows:

(8) $$Q[x,y,z,w]=\left\{ \begin{array}{l} 0\;\;\;\;\;x,y,z,w<Tv\;\;\\ 1\;\;\;\;\;\;x,y,z,w\geq Tv\; \end{array}\right.,$$

**Figure 11:**
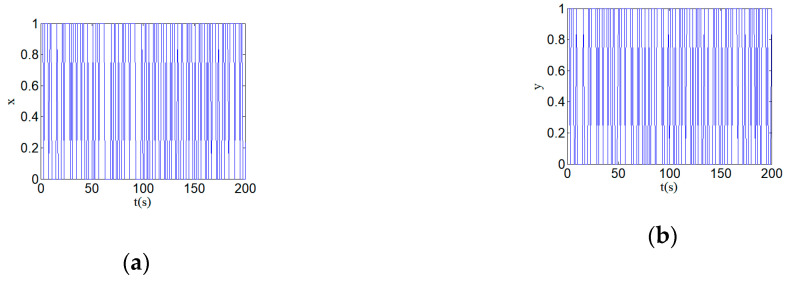

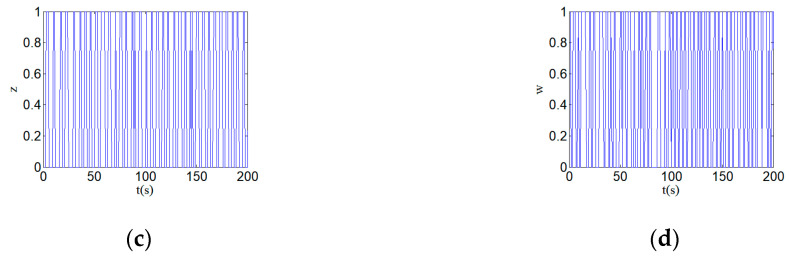
Quantized hyperchaotic time series: (**a**) quantized time series x; (**b**) quantized time series y; (**c**) quantized time series z; and (**d**) quantized time series w.

Here Tv=0, Q[⋅] is the quantized binary stream. The conversion value falls within the corresponding interval of the quantization function and gets 0 or 1, respectively. As chaotic signals [x,y,z,w] have good random statistical properties, the quantized stream (Q[⋅]) should have excellent statistical properties of equilibrium 0-1 ratio in theory. The streams after quantization of the time series x, y, z, and w are shown in Figure 11. Figure 11 shows the time series of the hyperchaotic system are quantized into 0-1 sequences.

### 4.3. NIST Test

The NIST SP 800-22 [50] random test package for stream cryptography (NIST random test) was provided by the National Institute of Standards and Technology. In order to verify the statistical performance of the quantized streams of the hyperchaotic system, NIST tests are carried out by using the test programs. The test package includes frequency test within a block, binary matrix rank test, non-overlapping template matching test, etc. These tests can be used to test binary sequences of an arbitrary length, generated by the pseudo-random number generator, which can be used to determine the non-randomness hidden in the stream. All of the test results are determined by P−value. If P<0.01, then the stream is not random. If P≥0.01, then the stream is considered random. In order to make the system get better randomness, this paper carries out NIST tests to prove that the random streams generated by the system can be used in the encryption application. Table 1 shows the test results. It can be seen that the quantized streams have good statistical characteristics and have passed the tests. Table 1 shows that the sequences generated by the new hypersystem have passed all the tests in statistical NIST tests. 

### 4.4. Permutation Entropy

The permutation entropy can be used to measure the complexity of time series. Permutation entropy is obtained by adding the permutation idea into the calculation of the complexity of sub-sequences. The algorithm is described as follows:

1. Define a time series x(1), x(2), …,x(N), m is the embedded dimension, τ is time delay.

2. Reconstruct the time series as X(i)=x(i), x(i+τ), …, x(i+(m−1)τ).

3. Increase and rearrange X(i). When $x(i+(j_{1}-1)\tau )\leq x(i+(j_{2}-1)\tau )\leq \ldots \leq x(i+(j_{m}-1)\tau )$, if the two values are equal, rearrange by subscript.

4. X(i) is redefined to $\left(j_{1},j_{2},\ldots ,j_{m}\right)$. Therefore, there will be m! permutations.

5. Define the probability distribution of all symbols as $p_{1}$, $p_{2}$, …, $p_{k}$, k≤m!.

6. The permutation entropy of the time series can be calculated by the following formula:

(9) $$H\left(m\right)=-\sum_{j=1}^{k}p_{j}\ln p_{j},$$

$p_{k}=1/m!$, that is to say that when the probability of each symbol is equal, then the stream has the maximum permutation entropy. To facilitate data analysis, H(m) will be normalized.

(10) 0≤H(m)/ln(m!)≤1,

The results of the permutation entropy test are shown in Table 2.

### 4.5. Approximate Entropy

Approximate entropy (ApEn) is used to measure the law of motion and unpredictability of a quantized time series, which is often used in nonlinear dynamics. It is characterized by the use of a non-negative number to represent the complexity of a time stream, which can reflect the possibility of new information in the stream. Therefore, the higher the approximate entropy is, the higher the complexity of the time series is. The algorithm description is shown as follows:

1. Define a time series U(1), U(2), …, U(N).

2. m is the length of the comparison vector. 

3. r is the measure of similarity.

4. Reconstruct the m dimension vector Y(1), Y(2), …,Y(N−m+1), and Y(i)=[U(i),U(i+1),…,U(i+m−1)].

5. When 1≤i≤N−m+1, calculate the number of vectors satisfying the following conditions:

(11) $$C_{i}^{m}(r)=\frac{1}{N-m+1}SUM[d(i,j)\leq r],$$

6. The function is defined as:

(12) $$\Phi^{m}(r)=\frac{1}{N-m+1}\sum_{i=1}^{N-m+1}\log(C_{i}^{m}(r)),$$

Here,$d(i,j)=\underset{a}{\max}\left|U(a)-U^{*}(a)\right|$. |U(a)| represents the element of the vector Y; d represents the distance between Y(i) and Y(j), whose value is determined by the maximum difference value of the corresponding element; j∈[1,N−m+1], j and i is allowed to exist in the case of equality.

From the above, the definition of Approximate Entropy (ApEn) can be obtained. In general, the value of the parameter m=2 or m=3 and r are determined by the actual application. Here r=0.2∗std, and std represents the standard deviation of the original time series. Normally, d(i,j)≤r. The more complex the time series is, the greater the corresponding approximate entropy is. The ApEn here is shown in Table 3, which means that the time series are of good unpredictability and can be used in nonlinear dynamics. 

## 5. Application in Image Encryption

### 5.1. Image Encryption Scheme

A digital image is represented by a two-dimensional matrix. Each element of the two-dimensional matrix represents the pixel value, and the coordinates of each element represent the location of the pixel. Permutation refers to taking the row and column of each element in the two-dimensional matrix of the image as the coordinate value of the pixel value, then using the encryption function to change the coordinate value of the pixel, thus changing the position of the individual pixel so that the original plaintext image cannot be recognized. Diffusion is to change the value of the pixels in the image, so as to change the statistical characteristics of the original image. Based on the principle of permutation and diffusion, the image encryption scheme here uses a low-dimensional chaotic system to obtain permutation, and high-dimensional hyperchaotic system to obtain diffusion, and generally achieves the effect of a two-step chaotic image encryption. Firstly, the sequence generated by the 1D Logistic chaotic system is used to construct the replacement table to transform the position of the original image to complete the permutation operation. Secondly, the stream cipher generated by the 4D hyperchaotic system proposed in this paper is used for diffusion operation to further ensure the security of image encryption.

**Figure 12:**
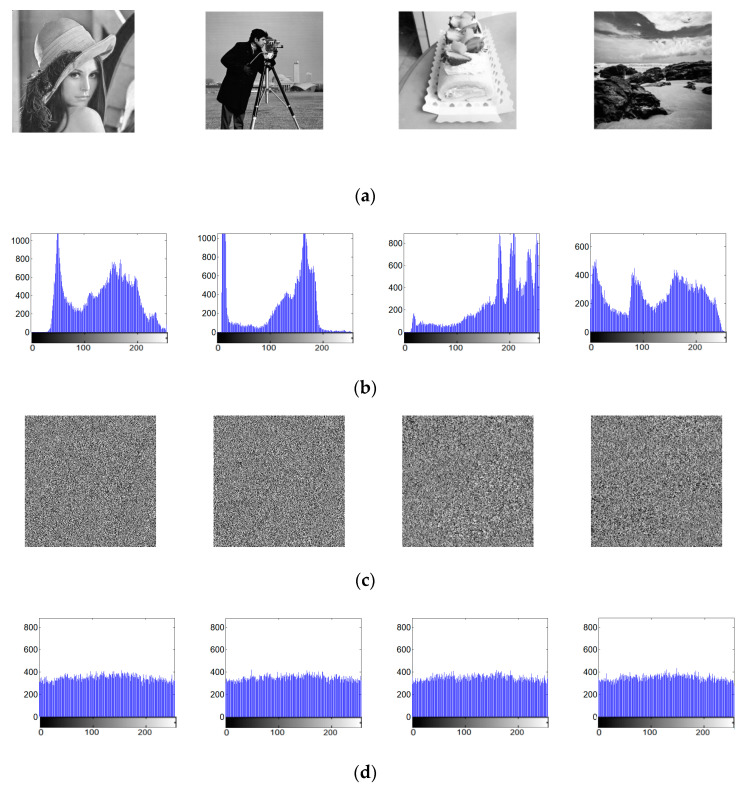
Comparison before and after image encryption of hyperchaotic stream: (**a**) original four images; (**b**) histograms of the four images; (**c**) encrypted four images; and (**d**) histograms of the encrypted four images.

Here, the hyperchaotic system stream is used to encrypt 256×256 Lena image, Cameraman image, Cake image, and Seaside image, respectively. The results of image encryption and security analysis are shown in Figure 12. Variance analysis can be used for testing the uniformity of the ciphered images. Through calculation, the histogram variance of the original Lena image is $6.2993\times 10^{4}$, and the histogram variance of the ciphered Lena image is 771.7529; the histogram variance of original Cameraman image is $1.1141\times 10^{5}$, and the histogram variance of the ciphered Cameraman image is 825.4153. It can be concluded that the histogram variances of the ciphered images are much smaller than those of the original images.

It can be seen that the histograms of the plain images in Figure 12 are very different. The different histograms mean that the distributions of the plain images are totally different. From the cipher images, it can be found that they are all random-like. The histograms of all the encrypted images are relatively flat and are very close to uniform distributions.

### 5.2. Correlation Coefficient Analysis

The correlation coefficient can be used to measure the degree of the correlation between two variables, with a value between −1 and 1. The Pearson correlation coefficient between two variables is defined as the quotient of covariance and standard deviation between two variables. The correlation coefficient, $r_{xy}$, is defined as follows:

(13) $$r_{xy}=\frac{\mathrm{cov}(x,y)}{\sqrt{D(x)\cdot D(y)}},$$

Here

(14) $$\mathrm{cov}(x,y)=\frac{1}{N}\sum_{i=1}^{N}\left(x_{i}-E(x))(y_{i}-E(y)\right),$$

(15) $$E(x)=\frac{1}{N}\sum_{i=1}^{N}x_{i},$$

(16) $$D(x)=\frac{1}{N}\sum_{i=1}^{N}{\left(x_{i}-E(x)\right)}^{2},$$

where x and y are two different image pixel values, N represents the number of all pixels, cov(x,y) represents the covariance, D(x) represents the variance of variable x, and E(x) represents the mean. The more observed variables are, the less the correlation coefficient is affected by the sampling error, and the more reliable the results are. The value range of the correlation coefficient is $r_{xy}\in \left[-1,1\right]$, and the closer $\left|r_{xy}\right|$ is to 1, the higher the correlation between the two variables is, and the closer the relationship between them is. $r_{xy}>0$ stands for positive correlation, $r_{xy}<0$ stands for negative correlation, and $r_{xy}=0$ stands for zero correlation for no correlation. 

**Figure 13:**
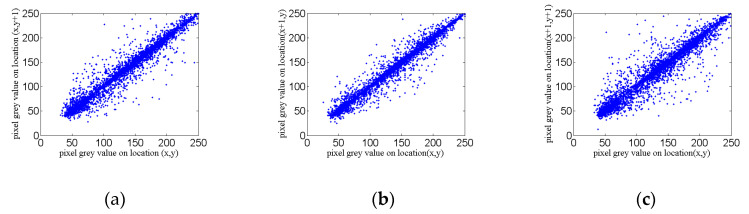
The Lena image correlation coefficient diagram before encryption: (**a**) horizontal correlation; (**b**) vertical correlation; and (**c**) diagonal correlation.

**Figure 14:**
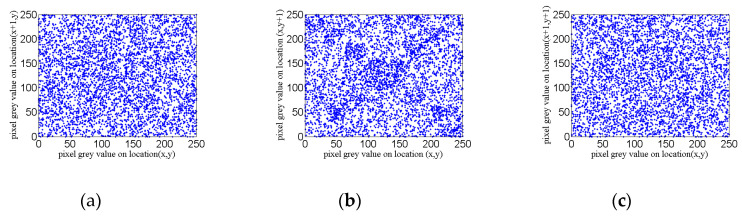
The Lena image correlation coefficient diagram after encryption: (**a**) horizontal correlation; (**b**) vertical correlation; and (**c**) diagonal correlation.

In this paper, the Lena image and its encrypted image are selected as the observation data, and a total of 5000 pairs of sampling points are used. The experimental results of correlation coefficients are shown in the Figure 13 and Figure 14. The comparison and analysis of the two groups of data are listed in Table 4. 

As can be seen from Figure 13 and Figure 14, the correlation coefficient diagrams before and after encryption are quite different. The correlation coefficient diagrams before encryption are of great correlation, and the diagrams after encryption are almost of no correlation.

In Table 4, the correlation coefficient of the Lena image before encryption is close to 1, which has a high correlation. The correlation coefficient of the encrypted graph is close to 0, indicating that there is almost no correlation, so it can well resist the corresponding statistical attack.

### 5.3. The Information Entropy

Information entropy can be used to measure the uncertainty of the randomly distributed gray value in an image. The definition of information entropy is shown as follows:

(17) $$H(m)=\sum_{i=1}^{N}p(m_{i})*\log\frac{1}{p(m_{i})},$$

where $p(m_{i})$ represents the probability of the sign $m_{i}$ occurring, and N represents the total number of $m_{i}$. Since the state of 256 grayscale images can reach $2^{8}$, the maximum value of information entropy, H(m), can be 8. In this paper, the information entropy of the Lena image and the photographer image is calculated and compared. The results can be seen in Table 5, which found that the entropy value of the encrypted image here is closer to the theoretical value 8. Therefore, the encryption scheme can effectively resist an information entropy attack.

### 5.4. Analysis of Differential Attack 

The attacker adds a small change to the system by changing some pixels in the image, so that the association between plaintext and ciphertext can be detected by observing the changes in the pre-encrypted and post-encrypted images. In general, to test the above, you can use the following two metrics to evaluate the encryption effect. One is pixel change rate (NPCR) [55], and the other is normalized mean change intensity (UACI) [55]. The two indicators are defined as follows:

(18) $$NPCR=\frac{\sum_{i,j}M(i,j)}{L_{1}\times L_{2}}\times 100\%,$$

(19) $$UACI=\frac{1}{L_{1}\times L_{2}}\sum_{i,j}\frac{C_{1}(i,j)-C_{2}(i,j)}{255}\times 100\%,$$

where, $C_{1}$ and $C_{2}$ are the values before and after the change of the pixel in the same position, and $C_{1}(i,j)$ and $C_{2}(i,j)$ represents the pixel intensity of the image (i,j) before and after the change. M(i,j) is a binary matrix of the same size as $C_{1}$ and $C_{2}$. If $C_{1}(i,j)\neq C_{2}(i,j)$, then M(i,j)=1, otherwise M(i,j)=0. In this paper, only one pixel value is changed, and the simulation results are shown in Table 6. The results showed that the NPCR value was close to 1, and the UACI value was close to 33.5% [38]. It shows that the encryption effect can resist some differential attacks. The test results are shown as follows:

### 5.5. Analysis of Plaintext Attack and Ciphertext Attack 

There are four typical types of attacks in an image encryption system, namely ciphertext only attack, chosen ciphertext attack, known plaintext attack, and chosen plaintext attack. The chosen plaintext attack is regarded as the most powerful attack among these attacks. If an image encryption system can resist chosen plaintext attack, it can be regarded to have the ability to resist the other three attacks [55]. From the differential attack analysis above, it is known that any small changes in the plain image will lead to a totally different cipher image. It means that the encryption system in this paper can resist differential attack, which is a typically chosen plaintext attack. The new hyperchaotic system has four parameters and presents different chaotic states and output sequences, with different initial values. The ciphered images are noise-like, and the corresponding histograms are close to uniform distributions. Therefore, the proposed image encryption scheme can resist against plaintext and ciphertext attacks.

### 5.6. Analysis of Noise Attack 

**Figure 15:**
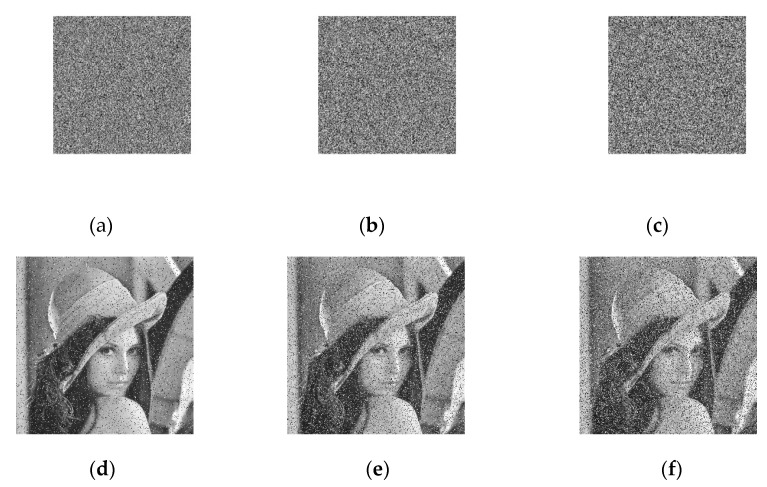
The ciphered images with different levels of noises and corresponding decrypted images: (**a**) Ciphered image with noise intensity 0.1; (**b**) ciphered image with noise intensity 0.2; (**c**) ciphered image with noise intensity 0.3; (**d**) decrypted image with noise intensity 0.1; (**e**) decrypted image with noise intensity 0.2; and (**f**) decrypted image with noise intensity 0.3.

The ciphered image is often changed by noise attack during the transmission process of the channel, making the receiver unable to decrypt correctly. Therefore, the anti-noise attack capability of an image encryption system is one of the criteria for measuring the anti-interference capability of the system. In order to test the anti-noise attack capability of the system, before decrypting the ciphered image, pepper and salt noises of different intensities were added to the ciphered image, and then the ciphered image with the noise was decrypted with the correct key. The ciphered image with pepper and salt noise and the decrypted image were respectively shown in Figure 15. Through comparative analysis, it can be seen that the encryption algorithm in this paper has better ability to resist anti-noise attacks.

### 5.7. Analysis of Exhaustive Attack

**Figure 16:**
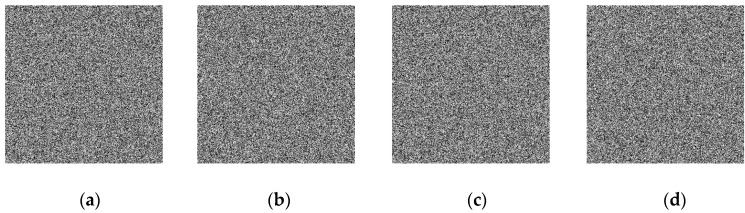
The decrypted images with minor key changes: (**a**) decrypted image with secret key $x_{0}+10^{-15}$; (**b**) decrypted image with secret key $y_{0}+10^{-15}$; (**c**) decrypted image with secret key $z_{0}+10^{-15}$; and (**d**) decrypted image with secret key $w_{0}+10^{-15}$.

In the analysis of exhaustive attack, the key space and key sensitivity can be analyzed, respectively. Firstly, the analysis of anti-exhaustive attack from the perspective of key space is discussed. The encryption key in this paper consists of two parts, one is the four system parameters of the hyperchaotic system a, b, c, and d; the other part is the four initial values of the hyperchaotic system $x_{0}$, $y_{0}$, $z_{0}$, and $w_{0}$. For the above four parameters and four initial values, if the calculation accuracy is $10^{-15}$, the total key space of the image encryption system is not less than $10^{120}$, so the encryption algorithm has enough key space to resist exhaustive attacks. Secondly, it discusses the analysis of anti-exhaustive attack from the perspective of key sensitivity. In order to test the key sensitivity of the image encryption system for the hyperchaotic system, four initial values of $x_{0}$, $y_{0}$, $z_{0}$, and $w_{0}$ were increased respectively, and the corresponding decrypted images were shown in Figure 16, under the condition that other keys do not change. It can be seen from the figure that the original image cannot be decrypted correctly even if the key is changed very slightly, so the image encryption algorithm has strong key sensitivity. In conclusion, the image encryption system has a good ability to resist exhaustive attacks.

## 6. Conclusions

In this paper, a new 4D hyperchaotic system was generated based on the Lorenz chaotic system. Through numerical calculation and computer simulation, the equilibrium point, local stability, and Poincare section of the hyperchaotic system were studied. It was found that there were periodic attractors, quasi-periodic attractors, and low-dimensional chaotic attractors in the hyperchaotic system. Then, the hyperchaotic system was normalized and discretized into binary random stream ciphers. Through NIST statistical test, permutation entropy, and approximate entropy analysis, it was found that this binary stream has good statistical performance. Finally, the binary stream generated by the hyperchaotic system was applied to the grayscale image encryption. It was concluded that the encryption scheme can resist statistical attack by the correlation coefficient and the information entropy analysis. From the analysis of differential attack, plaintext attack, ciphertext attack, noise attack, and exhaustive attack, it can be concluded that the encryption scheme can resist those attacks, which shows that the image encryption scheme in this paper can achieve a better encryption effect and resist most typical attacks.

## Figures and Tables

**Table 1:** NIST statistical tests.

Test	P−Value x	P−Value y	P−Value z	P−Value w	Result
Frequency Test	0.110561	0.251486	0.528546	0.228145	Success
Frequency Test within a Block	0.835247	0.142578	0.512879	0.627428	Success
Runs Test	0.759814	0.521473	0.715854	0.452842	Success
Test for the Longest Run of Ones in a Block	0.214189	0.204144	0.158728	0.157569	Success
Binary Matrix Rank Test	0.352471	0.521632	0.428745	0.638175	Success
Discrete Fourier Transform Test	0.644782	0.472575	0.284784	0.258741	Success
Non-Overlapping Template Matching Test	0.652417	0.418622	0.157525	0.784452	Success
Overlapping Template Matching Test	0.524718	0.514832	0.527865	0.518653	Success
Maurer’s “Universal Statistical” Test	0.195748	0.147258	0.285954	0.287695	Success
Linear Complexity Test	0.652149	0.625411	0.528765	0.318528	Success
Serial Test	0.058472	0.052148	0.078458	0.052145	Success
Approximation Entropy Test	0.024187	0.021458	0.025481	0.011285	Success
Cumulative Sums Test	0.421863	0.565281	0.627854	0.458654	Success
Random Excursions Test	0.524862	0.442389	0.458745	0.514865	Success
Random Excursions Variant Test	0.352874	0.328489	0.257841	0.258145	Success

**Table 2:** Permutation entropy value.

Time Series	*m*	*τ*	PE
x	3	1	0.6201
y	3	1	0.6548
z	3	1	0.5724
w	3	1	0.6017

**Table 3:** Approximate entropy value.

Time Series	*m*	r=0.2std	N	ApEn
x	2	0.1172	2048	0.7824
y	2	0.1036	2048	0.7653
z	2	0.1284	2048	0.7906
w	2	0.1165	2048	0.7819

**Table 4:** Correlation coefficient analysis of Lena Image and photographer image.

Direction	Horizontal	Vertical	Diagonal
Lena image before encryption	0.9842	0.6160	0.1969
Lena image after encryption	0.0043	−0.0230	−0.0027
[2] before encryption	0.9144	0.9545	0.9562
[2] after encryption	−0.0014	0.0028	0.0080
[38] before encryption	0.9254	0.9438	0.9325
[38] after encryption	0.0045	0.0012	0.0001
[45] before encryption	0.9577	0.9440	0.9126
[45] after encryption	−0.0082	0.0027	0.0030
[51] before encryption	0.9249	0.9593	0.9026
[51] after encryption	−0.0042	−0.0011	0.0029

**Table 5:** Information entropy value.

Image	InEn
Lena image	7.9978
[38]	7.9971
[52]	7.9965
[53]	7.9971
[54]	7.9851

**Table 6:** NPCR and UACI.

**Image**	**NPCR**	**UACI%**
Lena image	0.9961	33.42
